# Relationship of perceived environmental barriers and disability in community-dwelling elderly in Taiwan – a population-based study

**DOI:** 10.1186/1471-2318-14-59

**Published:** 2014-05-03

**Authors:** Wei-Chih Lien, Nai-Wen Guo, Jer-Hao Chang, Yu-Ching Lin, Ta-Shen Kuan

**Affiliations:** 1 Department of Physical Medicine and Rehabilitation, National Cheng Kung University Hospital, College of Medicine, National Cheng Kung University, Tainan, Taiwan; 2 Department of Physical Medicine and Rehabilitation, College of Medicine, National Cheng Kung University, Tainan, Taiwan; 3 Institute of Gerontology, College of Medicine, National Cheng Kung University, Tainan, Taiwan; 4 Institute of Behavioral Medicine, College of Medicine, National Cheng Kung University, Tainan, Taiwan; 5 Department of Occupational Therapy, College of Medicine, National Cheng Kung University, Tainan, Taiwan

**Keywords:** Perceived environmental barriers, Disability, Community-dwelling elderly

## Abstract

**Background:**

To identify the relationship between perceived environmental barriers and disability in community-dwelling elderly.

**Methods:**

Cross-sectional study in two community service centers in Tainan. We enrolled 200 community-dwelling residents, aged above 65 years, who had resided in the same community for at least 12 months. Basic activity of daily living (BADL) and instrumental activity of daily living (IADL) were assessed using the Hierarchy of Care Required (HCR). There were 59 participants in BADL disability and 109 in IADL disability. Perceived environmental barriers were assessed using the Craig Hospital Inventory of Environmental Factors (CHIEF). We used multinomial logistic regression to examine the relationship of perceived environmental barriers and disability.

**Results:**

The presence of perceived environmental barriers was related to BADL disability (OR = 4.39, 95% CI = 1.01-19.11) and IADL disability (IADL with difficulty in 1–2 tasks: OR = 9.93, 95% CI = 3.22-30.56; IADL with difficulty in more than 2 tasks: OR = 8.40, 95% CI = 1.83-38.51). The presence of physically/structurally perceived environmental barriers was related to BADL disability (OR = 4.90, 95% CI = 1.01-23.86) and IADL disability (IADL with difficulty in 1–2 tasks: OR = 4.61, 95% CI = 1.27-16.76; IADL with difficulty in more than 2 tasks: OR = 17.05, 95% CI = 2.82-103.30).

**Conclusions:**

Perceived environmental barriers are related to disability in community-dwelling elderly.

## Background

According to official statistics, the total number of people identified with disabilities in Taiwan was 1,117,518 in 2012, which was about 4.8% of the total population [1]. The average disability population among the elderly people in Taiwan (65 years and above) from the year 2003 to 2012 was 365,321.2 [1]. The increased prevalence of disabilities was linked to decreased quality of life [2] and increased health care costs [3,4].

It was pointed out by the sociologist Saad Nagi, “disability is the expression of a physical or a mental limitation in a social context” [5]. Several risk factors for the development of functional limitations have been identified, including lower extremity functional limitations, poor self-perceived health, low level of physical activity, depression, cognitive impairment, disease burden (co-morbidity), increased and decreased body mass index, low frequency of social contacts, smoking and vision impairment [6]. According to Lewin’s person-environment (PE) fit model, the degree to which elderly individuals can deal with disabilities and environmental challenges has been shown to determine how well they will function in their real-life settings [7,8].

Influential work that conceptualized the role of both the individual home environment and individual performance was published by Lewin in his person-environment (PE) fit model [9]. The PE fit model is composed of two interactive components: the *person component* and the *environment component*. In this model, the *person component* is defined as a set of competencies, including biological health, cognitive function, and sensory and motor skills. The *environment component* is defined in terms of demands, which are expressed as environmental “press” (i.e. the strength with which the environment demands a response from the person) [10].

The initial model was developed to explain the interaction between individuals and their home environment [9] and then was expanded to the neighborhood environment [11]. In extending this concept to the public health realm, the ideal amount of “press” would be different in an individual’s home as compared to the neighborhood environment. This is also agreed with the International Classification of Functioning, Disability and Health (ICF) developed by the World Health Organization [12]. The ICF model addresses functioning at three levels: 1) the body (mental, physiologic, or anatomic structures or functions), 2) the person (performance or accomplishment of an activity), and 3) the society (participation in life situations).

In recent years, the ICF model has been applied in monitoring of functioning in elderly in several studies [13,14]. According to the ICF model, the interaction between the individual and the environment can play a key role in determining the level of participation in the society [15]. The gathering and provision of holistic information related to environmental barriers for community-dwelling elderly can stimulate researchers and policy makers to enact changes that will reduce demands on the elderly with functional limitations and will also increase support for promoting community-level functioning in the elderly [13,14].

In the case of the elderly, environmental barriers are related to disabilities [16], fall risk [17,18], fall-related fractures [19], less commuting [20] and being homebound [21]. Many studies examining the relation between the environment and health have relied on secondary data sources to obtain objective characteristics of the environment through proxy measures, such as population density [22], land use diversity [22], block size [23], air pollution [24], urban–rural gap [25], or extremely seasonal temperatures [24]. These individual records, which were simple aggregates of individual or land use characteristics within areas, were often too large to meaningfully obtain the actual environments faced by the elderly when they walk outside their home and might in addition be ecologically inaccurate.

In this research, we examined the relationship between perceived environmental barriers and disability. We hypothesize perceived environmental barriers to be associated with disabilities related to basic activities of daily living (BADL) and also to instrumental activities of daily living (IADL).

## Methods

A cross-sectional design utilizing the face-to-face administration of the study instruments and direct observation were used for this study. We enrolled cases from two community service centers in Tainan, Taiwan. Of the 213 local residents who regularly came to these two community service centers to have their blood pressure taken in 2012, 204 community-dwelling residents, aged above 65 years, who had resided in the same community for at least 12 months were selected to participate in this study. Exclusion criteria included being diagnosed as having dementia and low scores (below 8 points) on the Chinese version of Short Portable Mental State Questionnaire (SPMSQ) (a 10-item questionnaire with total score ranged from 0 to 10 and a total score of eight and above represented intact cognitive functioning) [26]. The study was administered by one well-trained physician from December 2012 to August 2013. All of the sampled subjects were invited to participate when they came to their community service centers. Of the 204 subjects, 202 agreed to participate in the study. Two subjects were excluded due to SPMSQ below 8 points. As a result, the final case number was 200. It took 30–50 minutes to complete one subject. Approval for this study was provided by the Institute Review Board of National Cheng-Kung University Hospital, College of Medicine, National Cheng Kung University (Approval No: B-ER-101-184). Prior to inclusion in the study, the participants were informed of the study content and given the opportunity to decline participation.

### Perceived environmental barriers

Perceived environmental barriers were assessed by the Craig Hospital Inventory of Environmental Factors (CHIEF) [27]. The 25-item CHIEF is a tool commonly used to assess environmental barriers. We rated each item based on two scales. First, a 5-point frequency scale (0: never, 1: less than monthly, 2: monthly, 3: weekly, 4: daily) was used to indicate the frequency with which barriers were encountered. Secondly, a 2-point magnitude scale (1: a little problem, 2: a big problem) was used to indicate the extent of the problem a barrier typically presents. A frequency-magnitude product score (score range 0–8), calculated as the product of the frequency score and the magnitude score, indicated the overall impact of the barrier. The frequency-magnitude product score of different individual items were summed to create total CHIEF score and averaged to create mean of the subscale scores (Physical/Structural, Attitudes/Support, Services/Assistance, Policies, and Work/School). CHIEF has been used to investigate perceived environmental barriers in people with spinal cord injuries [28,29], traumatic brain injuries [30], and stroke [31,32].

After permission for use was obtained from the original authors of CHIEF, cross-cultural adaptation of the original questionnaire was performed according to recent guidelines [33]. First of all, the CHIEF was translated from English into Chinese by two bilingual persons. One of them had prior knowledge of the questionnaire. Both Chinese translations were compared with each other for inconsistencies. A consensus was reached by the synthesis of two translations after discussion. The CHIEF was then translated back into English by a Chinese-American with familiarity in both English and Chinese who had not seen the text previously. Consequently, a few changes were made. Next, a group meeting was held. The group meeting consisted of three individuals (a psychologist, an occupational therapist, and a physiatrist), who are competent in both Chinese and English. In the group meeting, we deleted six questions mentioning work and school because there are few people aged above 65 years old still in school or still working according to the official statistics [34] and regulations. We added four questions to compare with the original questions related to “community” in Chinese, and deleted one question about service in the “community”. Finally, two community-dwelling elderly individuals completed the CHIEF and provided feedback on the translated questionnaire. Minor changes to the questionnaire were then made to yield the pre-final Chinese version of CHIEF for community-dwelling elderly.

We enrolled 200 community-dwelling elderly for the measurement of construct validity. Demographic data are shown in Table 1. There were four questions with values of less than 0.3 in the corrected item-total correlation, indicating that these questions did not correlate well with the scale overall. After dropping these four questions, the value of Kaiser-Meyer-Olkin measure of sampling adequacy was 0.863. Cronbach’s Alpha was .896, and four factors could explain 67.5% of the variation in CHIEF. The four factors formed logical constructs, which were labeled as subscales called “services and assistance barriers,” “attitude and support barriers,” “physical and structural barriers,” and “policy barriers.” Each subscale in CHIEF for community dwelling elderly in Taiwan (18 questions) contained two to six questions, which were averaged to create mean of the subscale scores. The final version of CHIEF for the Community-dwelling Elderly in Taiwan (CHIEF-CET) was the result.

**Table 1:** Subject characteristics and the related mean of the subscale scores of CHIEF-CET

**Subject characteristics**	**No. (%)**	**Service/Assistance**	**Attitude/Support**	**Physical/Structural**	**Policy**
Age(y)					
65–74	107 (53.5%)	0.38	0.49	0.40	0.29
75 and older	93 (46.5%)	0.80	0.88	0.75	0.66
Gender					
Female	117 (58.5%)	0.58	0.62	0.58	0.47
Male	83 (41.5%)	0.57	0.74	0.55	0.44
Education					
Literate	149 (74.5%)	0.51	0.59	0.50	0.37
Illiterate	51 (25.5%)	0.76	0.90	0.75	0.72
Living arrangement					
Live with spouse/kids or others	169 (84.5%)	0.49	0.62	0.48	0.38
Live alone	31 (15.5%)	1.03	0.95	1.05	0.90
Financial status					
Non-lower ranking family	168 (84%)	0.53	0.65	0.51	0.37
Lower-ranking family	32 (16%)	0.83	0.79	0.88	0.94
Self-rated health					
Good-very good	28 (14%)	0.12	0.03	0.01	0.04
Very poor-fair	172 (86%)	0.65	0.78	0.66	0.53
GDS					
0–5	165 (82.5%)	0.57	0.66	0.53	0.47
6-7	35 (17.5%)	0.62	0.72	0.75	0.41
TUG					
0-10secs	110 (55%)	0.36	0.41	0.29	0.23
>10secs	90 (45%)	0.84	1.00	0.90	0.74
BADLs					
No disability	141 (70.5%)	0.48	0.50	0.42	0.29
Disability	59 (29.5%)	0.80	1.09	0.91	0.87
IADLs					
No disability					
With difficulty in 1–2 tasks	51 (25.5%)	0.84	0.94	0.71	0.48
With difficulty in more than 2 tasks	58 (29%)	0.79	1.11	0.91	0.89

### Basic activity of daily living (BADL) and Instrumental activity of daily living (IADL)

BADL and IADL were assessed using the Hierarchy of Care Required (HCR) [35]. HCR combines three subscales, including BADLs, IADLs, Cognition and Emotion. Every subscale has six activities, and each is divided into five levels. The six BADL tasks include feeding, hygiene (including grooming & bathing), clothing, sphincter control, transferring, and mobility. The six IADL tasks include making a meal, shopping, telephoning, financial management, taking medication, and transportation. The six cognitive and emotion (C&E) tasks include comprehension, expression, social interaction, memory, meta-emotion, and reality-testing. The item responses for each task were scored 1 to 5 (1, no need for help; 2, intermittent; 3, supervision; 4, need assistance all the time; 5. unable to do). The Cronbach’s α was 0.88-0.98, and 67%-86% of the variation in the HCR subscales was explained. In the case of the community-dwelling elderly in Taiwan, the Cronbach’s α has been found to be 0.68-0.98 [35].

Participants with no need for help in doing both BADL and IADL tasks were classified as no disability. Participants who reported difficulty in at least one BADL task were classified as BADL disability. Participants who reported difficulty in at least one IADL task were classified as IADL disability. IADL disability was dichotomized into IADL with difficulty in 1–2 tasks and IADL with difficulty in more than 2 tasks [36].

### Mobility limitation

Mobility limitation was assessed using the Time Get Up and Go test (TUG) which consists of a timed sequence including rising from a chair, walking three meters, turning, and returning to sit in the chair [37]. This test has been used extensively in geriatric medicine to examine balance, gait speed, and the functional ability that would be required for the performance of basic activities of daily living in older people [38]. The time needed for TUG in healthy community-dwelling elderly aged between 60 to 79 year-old has been found to be about 7 to 9 seconds [39]. The cut-off time separating non-fallers and fallers has typically varied from 10 to 32.6 seconds [40]. Optimal cutoff values for separating physically abled from disabled older men and women in Taiwan, respectively have been determined to be 8.77 seconds and 9.12 seconds [41]. Sensitivity was found to be 69.0% and 63.2%, and specificity was 76.9% and 76.6%, respectively.

### Personal factors

The personal factors used in this study included age, gender, education, living arrangements, financial status, and self-perceived health status. We dichotomized the personal factors as the following: age (65–74 years old/75 years old and above), gender (male/female), education (illiterate/literate), living arrangements (living alone/not living alone), financial status (lower-ranking family: average personal income in a family per year < 6400 US/non lower-ranking family > 6400 US), SRH (Likert scale from very poor, poor to fair: 0-3/from good to very good: 4–5) and TUG (<=10 seconds/>10 seconds). The Chinese translated Geriatric Depression Scale was used to assess the mood of the elderly people under consideration in this study [42-44]. The scores on the 15-question version (each question with a ‘yes’ answer receives 1 point) range from 0 to 15, with a score of 4/5 [43] or 5/6 [42] being the cutoff point for the categorization of “depressive symptoms” (“yes” or “no”) in several studies. The cut-off point in this study was 5/6.

### Statistical analysis

HCR BADL and IADL subscales associated with perceived environmental barriers were calculated with a multinomial logistic regression, using the total CHIEF-CET score and its four subscales as dichotomous dependent variables. The multinomial logistic regression was conducted to control for possible effects of other confounding factors. We then put the four subscales of CHIEF-CET into the multinomial logistic regression to measure the odds ratio (OR) of each subscale after adjusting for age, gender, education, living arrangement, financial status, GDS, SRH and TUG. Crude and adjusted odds ratios were performed to measure the associations between BADL and perceived environmental barriers, between IADL difficulty in 1–2 tasks with perceived environmental barriers, and between IADL difficulty in more than 2 tasks with perceived environmental barriers. Ninety five percent confidence intervals (CIs) not including 1 or p < .05 were regarded to be statistically significant. All analyses were performed using the Statistical Package of Social Science (SPSS) version 12.0.

## Results

We collected data from a total of 200 community-dwelling elderly individuals. The mean age was 74.9 ± 6.9 years old. Subject characteristics and the related means of the domain scores for the CHIEF-CET are shown in Table 1.

Results for the multinomial logistic regression analysis appear in Table 2 for perceived environmental barriers and in Table 3 for each subscale. The results indicate that the presence of perceived environmental barriers was related to BADL disability (adjusted OR = 4.39, 95% CI = 1.01-19.11). The presence of perceived environmental barriers was also related to IADL disability (IADL with difficulty in 1–2 tasks: adjusted OR = 9.93, 95% CI = 3.22-30.56; IADL with difficulty in more than 2 tasks: adjusted OR = 8.40, 95% CI = 1.83-38.51). The presence of physically/structurally perceived environmental barriers was related to BADL disability (adjusted OR = 4.90, 95% CI = 1.01-23.86). The odds ratio of BADL disability (OR = 4.90) indicated that the presence of physically/structurally perceived environmental barriers increased the odds of BADL disability by 3.90 times. The presence of physically/structurally perceived environmental barriers was related to IADL disability (IADL with difficulty in 1–2 tasks: adjusted OR = 4.61, 95% CI = 1.27-16.76; IADL with difficulty in more than 2 tasks: adjusted OR = 17.05, 95% CI = 2.82-103.30). The odds ratio of IADL with difficulty in 1–2 tasks (OR = 4.61) indicated that the presence of physically/structurally perceived environmental barriers increased the odds of IADL with difficulty in 1–2 tasks by 3.61 times. The odds ratio of IADL with difficulty in more than 2 tasks (OR = 17.05) indicated that the presence of physically/structurally perceived environmental barriers increased the odds of IADL with difficulty in more than 2 tasks by 16.05 times.

**Table 2:** Associations between perceived environmental barriers and BADL/IADL disability

	**BADL disability**		**IADL with difficulty in 1–2 tasks**		**IADL with difficulty in more than 2 tasks**	
	**Crude OR(95% CI)**	**Adjusted OR(95% CI)**	**Crude OR(95% CI)**	**Adjusted OR(95% CI)**	**Crude OR(95% CI)**	**Adjusted OR(95% CI)**
Perceived environmental barriers						
No(ref)	1.00	1.00	1.00	1.00	1.00	1.00
Yes	13.04***(3.89-43.70)	4.39*(1.01-19.11)	12.83***(4.66-35.32)	9.93***(3.22-30.56)	25.57***(7.44-87.87)	8.40**(1.83-38.51)

*p < .05; **p < .01; ***p < .001.

The definition of reference group was participants who perceived no environmental barriers (CHIEF-CET score = 0).

Greater odds ratios (OR) represent higher likelihood of disability. Adjusted for age, gender, education, living arrangement, financial status, GDS, SRH and TUG.

**Table 3:** Associations between each subscale of the perceived environment barriers and BADL/IADL disability

	**BADL disability**		**IADL with difficulty in 1–2 tasks**		**IADL with difficulty in more than 2 tasks**	
	**Adjusted OR**	**95% CI**	**Adjusted OR**	**95% CI**	**Adjusted OR**	**95% CI**
Service/Assistance						
No(ref)	1.00	0.24-6.78	1.00	0.17-4.64	1.00	0.12-4.81
Yes	1.27		0.90		0.75	
Physical/Structural						
No(ref)	1.00	1.01-23.86	1.00	1.27-16.76	1.00	2.82-103.30
Yes	4.90*		4.61*		17.05**	
Attitude/Support						
No(ref)	1.00	0.26-3.98	1.00	0.84-12.99	1.00	0.33-6.51
Yes	1.02		3.30		1.46	
Policies						
No(ref)	1.00	0.51-3.39	1.00	0.60-5.17	1.00	0.64-6.43
Yes	1.32		1.76		2.03	

*p < .05; **p < .01.

In the subscale of Service/Assistance, the definition of reference group was participants who perceived no Service/Assistance environmental barriers (services and assistance barriers score = 0). In the Physical/Structural subscale, the definition of reference group was participants who perceived no Physical/Structural environmental barriers (physical and structural barriers score = 0). In the subscale of Attitude/Support, the definition of reference group was participants who perceived no Attitude/Support environmental barriers (attitude and support barriers score = 0). In the subscale of Policies, the definition of reference group was participants who perceived no Policies environmental barriers (policy barriers score = 0).

Greater odds ratios (OR) represent higher likelihood of disability. Adjusted for age, gender, education, living arrangement, financial status, GDS, SRH and TUG.

## Discussion

In this study, a cultural adaptation of the CHIEF questionnaire was performed to facilitate measurement of environmental barriers among community-dwelling elderly people in Taiwan. The mean value of CHIEF-CET total score was 0.59, which is higher than the mean value of the CHIEF total score previously reported for the elderly in Korea (0.52) [31]. The mean of the subscale scores were as follows: Services/Assistance = 0.58, Physical/Structural = 0.67, Attitude/Support = 0.57, and Policies = 0.46. Among the four subscales, the Physical/Structural subscale showed the highest score, just as has been the case for patients who have experienced strokes [31,32], spinal cord injuries [27] and traumatic brain injuries [27]. In Tainan City, which is a densely populated city with a relative lack of age-friendly buildings and facilities, it was not surprising that elderly individuals with functional limitations might encounter some physical and structural environmental barriers that restrict their social participation.

The focus of this analysis was an examination of relationships between difficulty with BADL/IADL and perceived environmental barriers among the elderly living in Tainan, Taiwan. The presence of difficulty related to BADL/IADL was associated with perceived environmental barriers after controlling for possible confounding factors. Our results were in accordance with those of previous studies in which perceived environmental barriers were found to be significantly related to activities of daily life but not to age, gender, or the occurrence of strokes in Korean community-dwelling elderly individuals [31].

We also added the four subscales into multinomial logistic regression in order to examine the differences in these four subscales. Physical/Structural environmental barriers were related to BADL and IADL with difficulty. Attitude/Support and policies environmental barriers were related to BADL and IADL with difficulty only in the descriptive analyses and not in the multivariate analyses. Service/Assistance environmental barriers were related to BADL with difficulty only in the descriptive analyses and not in the multivariate analyses. Different associations between the subscales of perceived environmental barriers and disability might be explained by compensatory strategies in elderly individuals.

*Compensatory strategy* has been defined as “a way of achieving a result that is adopted frequently in the face of physical impairment or limitation and under usual conditions.” [45] In Taiwan, three types of compensatory and coping strategies for disabled elderly were identified: (1) seeking support related to greater adaptation to difficulty in terms of family relationships; (2) avoidance of increases in the difficulty of adapting in the health-care, domestic environment and psychological distress dimensions; and (3) acceptance of reductions in the difficulty related to adapting to disability [46]. Seeking support related to greater adaptation to difficulty in terms of family relationships might attenuate the association between support environmental barriers and disability [47].

Overall, the strength of this study was that it provided a well-characterized environmental assessment that links the environment to estimates of BADL and IADL disability. Therefore, instead of using the CHIEF-CET, an effort was made to determine whether the Physical/Structural subscale is a more sensitive and convenient predictive tool for ADL disability in the community-dwelling elderly in Taiwan. Based on the results, the government should pay more attention to age-friendly buildings and facilities in order to reduce demands on elderly members of the population suffering from functional limitations, and the government should also promote community-level functioning in the elderly.

### Limitations

There were some limitations in this study. First, because this was a cross-sectional study, a causal effect of perceived environmental barriers on disability cannot be claimed. Future research involving longitudinal studies of perceived environmental barriers and objective environmental barriers with disability are warranted.

Next, pathology was not included in this study. However, in recent years, a hierarchical relationship between pathology, mobility, IADL and BADL limitations has been verified both by cross-sectional research [48] and longitudinal research [49,50]. Thus, a majority of elderly individuals with co-morbidities develop disabilities in the mobility domain first, followed by IADL and eventually, BADL. The main pathway from pathology to disability is through mobility limitations among the elderly. Although we didn’t measure co-morbidities, we did measure lower body functional limitations.

Third, this study was based on 200 subjects from two community service centers. The relatively small population size might have limited the statistical power of the detected associations. Finally, it might be difficult to justify generalizing the results of this study for the entire population of Taiwan without any further information. However, these two communities are typical urban communities in Taiwan. The results might be suitable for application to other urban communities in Taiwan. In Taiwan, the majority of communities are located in urban areas. Hence, the results are still valuable for policy makers in Taiwan.

## Conclusions

Perceived environmental barriers are related to disability in community-dwelling elderly. These results underscore the importance of perceived environmental barriers on activity limitation and participation restriction, especially when dealing with an aging population that is likely to exhibit an increased number of disabilities over the coming years.

## Competing interests

The authors declare that they have no competing interests.

## Authors’ contributions

WCL, NWG, JHC, YCL and TSK designed the present study. WCL and TSK enrolled the subjects, extracted the data, performed the statistical analysis, and wrote the original draft. WCL, NWG, JHC, YCL and TSK revised the draft critically and approved the final manuscript.

## Pre-publication history

The pre-publication history for this paper can be accessed here:

http://www.biomedcentral.com/1471-2318/14/59/prepub
